# Comparative genomic analysis of *Sinorhizobium meliloti* LPU88: plasmid diversity and conjugative mechanisms

**DOI:** 10.1128/aem.01996-25

**Published:** 2026-05-06

**Authors:** Constanza Rey, Andrés M. Toscani, Juliet F. Nilsson, Lucas G. Castellani, Ramiro E. Rocco Welsh, Abril Luchetti, Tobias Busche, Jörn Kalinowski, Gonzalo Torres Tejerizo, Mariano Pistorio

**Affiliations:** 1 Instituto de Biotecnología y Biología Molecular (IBBM), CCT-CONICET-La Plata, Departamento de Ciencias Biológicas, Facultad de Ciencias Exactas, Universidad Nacional de La Plata 172355 , La Plata, Argentina; 2 Institute for Multidisciplinary Research in Applied Biology (IMAB), Universidad Pública de Navarra (UPNA) 16756, Pamplona, Spain; 3 Microbial Genomics and Biotechnology, Center for Biotechnology, Bielefeld University 117229, Bielefeld, Germany; The University of Tennessee Knoxville, Knoxville, Tennessee, USA

**Keywords:** plasmid, conjugation, *Sinorhizobium meliloti*

## Abstract

In this study, we present a comprehensive genomic and comparative analysis of *Sinorhizobium meliloti* strain LPU88, highlighting the structure, function, and evolutionary dynamics of its plasmids. The complete genome sequencing revealed five replicons: a chromosome, two megaplasmids (pSymA-like pSmeLPU88c and pSymB-like pSmeLPU88d), and two accessory plasmids (pSmeLPU88a and pSmeLPU88b). Furthermore, the genome of LPU88 harbored a rich repertoire of mobile genetic elements, diverse replication modules, and unique gene clusters, reflecting its dynamic architecture. Strain LPU88 contained diverse conjugation systems distributed across its plasmids. Comparative analyses with other *S. meliloti* and *Sinorhizobium medicae* strains demonstrated the heterogeneous distribution of conjugative and regulatory elements, indicating variable evolutionary pressures among these plasmids. Besides, the mobilization of the pSymA-like plasmid pSmeLPU88c was mediated by a mating pair formation system encoded on the accessory plasmid pSmeLPU88a, reflecting the intricate mechanisms and evolutionary dynamics of horizontal gene transfer mediated by plasmids in *Sinorhizobium*. By integrating genomic sequencing, functional annotation, and comparative approaches, this work establishes LPU88 as a valuable model strain for understanding plasmid diversity, horizontal gene transfer, and symbiotic efficiency in rhizobia.

**IMPORTANCE:**

Rhizobia are soil bacteria that establish symbiotic associations with legumes, converting atmospheric nitrogen into ammonia through biological nitrogen fixation, while the host provides nutrients. Among them, *Sinorhizobium meliloti* is one of the best-studied species. In this work, we compared the complete genomes of *S. meliloti* strains, including the laboratory model strain LPU88, with a particular focus on pSymA plasmids. Previous studies proposed that the pSymA plasmid could have been acquired through horizontal gene transfer. Analysis of their conjugation machinery revealed that all pSymA plasmids harbor a type II conjugation system, although in many cases the regulatory circuit required for activation was absent. In LPU88, we identified and characterized multiple conjugation systems, offering new insights into horizontal gene transfer in *S. meliloti*. Understanding these processes is essential for clarifying rhizobial evolutionary dynamics, improving the stability and efficiency of symbiotic interactions, and promoting their use as bioinoculants in sustainable agriculture.

## INTRODUCTION

Rhizobia are soil bacteria capable of establishing a symbiotic relationship with legume plants. In this relationship, rhizobia reduce molecular atmospheric nitrogen to ammonia through biological nitrogen fixation, and legumes supply nutrients to bacteria (1). This process has significant ecological and economic importance since it avoids the use of contaminating and expensive nitrogenous fertilizers (2). A common feature of rhizobia species belonging to the genera *Rhizobium* and *Sinorhizobium* is the presence of large amounts of extrachromosomal DNA in plasmids. These plasmids vary greatly in number and size, and many of them have important roles in the association of rhizobia with legume plants (3, 4). One of the most studied rhizobia is *Sinorhizobium meliloti*, which establishes a symbiosis with legumes of the genera *Medicago* and *Melilotus*, including *Medicago truncatula* and *Medicago sativa* (alfalfa) (5). All sequenced genomes of *S. meliloti* strains contain three large replicons: the chromosome and two large extrachromosomal replicons (megaplasmids). Additionally, some strains harbor a variable number of accessory plasmids (6–10). The megaplasmids are designated symbiotic plasmids (pSyms) since they contain genes that are essential for the establishment of a complete symbiotic state. Along with symbiotic genes, several non-symbiotic genes are present in pSyms. The *S. meliloti* pSymA-like plasmid carries the genes required for nodulation (*nod*), nitrogenase (*nif*), and nitrogen fixation (*fix*) (11). In addition, pSymA-like plasmids carry genes involved in nitrogen and carbon metabolism, transport, stress responses, and resistance that could give *S. meliloti* an advantage in certain environmental situations (12–14). pSymB carries genes that code for solute uptake systems along with enzymes involved in both polysaccharide biosynthesis (essential for the early stages of symbiosis) and catabolic activities (6, 15). pSymB also carries genes that are essential for cell viability, named housekeeping genes (13), which is why it has been designated a chromid (16). In contrast, no essential genes have been mapped to any of the already sequenced pSymA-like plasmids. Moreover, Oresnik et al. (17) and later diCenzo et al. (13) were able to cure this plasmid, without affecting the viability of the derivative strain. On the other hand, pSymB was only cured after duplicating essential genes in the chromosome (13). Accessory plasmids are those that are not necessary for the establishment of a complete symbiotic state (4). In most cases, their function is still unknown, and their size varies greatly. It has been proposed that some regions of these plasmids may be involved in genetic rearrangements (18, 19), leading to the presence of repeated sequences, deletions, or even plasmid co-integration, contributing to their structural plasticity and dynamic genomic content. Furthermore, many of these plasmids encode functions that provide the cell with an adaptive advantage in certain environments. Like symbiotic plasmids, many non-symbiotic plasmids may carry genes that enable the bacteria to utilize different organic carbon sources (20, 21). In other cases, genes in non-symbiotic plasmids hinder the symbiotic development (22).

The most common plasmid family present in rhizobia is the *repABC*-type, whose structure and mechanism of replication have both been largely studied, followed by the evolutionarily related *repC* family of replicons (23–25). Numerous studies have demonstrated that both symbiotic and accessory plasmids can be transferred to other microorganisms via conjugation. Conjugative systems are composed of DNA transfer and replication (Dtr) and mating pair formation (Mpf) systems (26). The Dtr system contains the proteins responsible for DNA processing, while the Mpf system involves the proteins that form the conjugative pore (27). Rhizobial plasmids exhibit four distinct groups of conjugative systems, classified based on the characteristics of the Dtr and the Mpf systems’ pores (27–29). Yet, detailed regulatory mechanisms have only been elucidated for specific cases (30–32). Among these, one system is TraR dependent, while another involves the *rctA/rctB* genes (30–34). The latter system is involved in the regulation of pSymA conjugal transfer in *S. meliloti* strain 2011 (31, 34). Specifically, *rctA* encodes a transcriptional repressor of the Mpf system. The transcription of *rctA* is positively auto-regulated, but this regulation is attenuated when *rctB* is overexpressed, with RctB functioning as a conjugation activator (31, 35). Two additional genes, *rctR* and *rctC*, have been identified within this regulatory network (34). It has been proposed that the expression of *rctR*, which represses conjugation, is inhibited by an unidentified environmental signal (34). Nonetheless, recent evidence from other laboratories, involving *Rhizobium leguminosarum* and *Rhizobium etli* (27, 36), as well as our own research on *S. meliloti* and *Rhizobium favelukesii* (37–40), suggests the presence of new regulatory mechanisms. These mechanisms involve other genes and indicate that both genetic and environmental contexts play critical roles in the regulation of conjugation.

In previous studies, Luchetti et al. (41) characterized the replication and transmissibility properties of accessory plasmids in strain *S. meliloti* LPU88. In the plasmid pSmeLPU88b, two replication modules were identified: one belonging to the *repABC* system replicons and the other to the *repC* family of replicons (29, 41). Through functional studies of both replication origins, it was demonstrated that the *repABC* system is more stable than the *repC* system (41). Furthermore, the plasmid pSmeLPU88b was mobilizable when helper functions were provided by plasmid pSmeLPU88a, forming a binary conjugation system (binary conjugal system) (42). Despite these findings, the characteristics of mating pair formation and replication modules for plasmid pSmeLPU88a have not been described.

To gain further insight into the conjugative characteristics of the binary system of plasmids pSmeLPU88a/pSmeLPU88b, we sequenced the genome of strain LPU88 and performed a deep characterization of the conjugative systems present in this strain. Here, we present a summary of the general features of *S. meliloti* strain LPU88, along with a description of its genome sequence and annotation, and a comparison of symbiotic clusters, plasmid replication systems, and the presence of mobile genetic elements with other complete *S. meliloti* genomes. Furthermore, we identified three Mpf systems and four Dtr systems. Notably, an additional Mpf and Dtr system was found on plasmid pSmeLPU88c (pSymA-like), and the functionality of this Mpf system was also analyzed.

## MATERIALS AND METHODS

### Bacterial strains, plasmids, and growth conditions

The strains and plasmids used in this work are listed in Table S1 at https://doi.org/10.24215/10915datasetDBEERZ. *Escherichia coli* strains were grown in liquid or solid lysogeny broth medium at 37°C, supplemented with the corresponding antibiotic. *S. meliloti* strains were grown at 28°C in tryptone yeast (TY) medium (43). We used 15 g/L of agar for solid media. Antibiotic concentrations used were as follows (μg/mL): kanamycin, 50, and gentamicin (Gm), 10, for *E. coli*; tetracycline, 5; streptomycin (Sm), 400; neomycin (Nm), 120; Gm, 50; rifampicin, 200; and chloramphenicol (Cm), 20, for *S. meliloti*.

### DNA sequencing and bioinformatics tools

The genome of the LPU88 strain was sequenced using a combination of Illumina and Oxford Nanopore sequencing technologies. Illumina sequencing was performed at SNPsaurus (Eugene, OR, USA) with an Illumina HiSeq 4000. The sequencing was carried out using Illumina paired-end libraries with 2 × 150 bp reads to reach ca. 60× read depth. Additionally, an Oxford Nanopore Technologies (ONT) library was constructed through the MinION platform to obtain the complete genomic structure. The genome assembly was first performed using ONT sequences with FLYE 2.9.1-b1780 (44), followed by an improvement with PILON 1.22 (45) using Illumina sequences. The genome was annotated using the NCBI Prokaryotic Genome Annotation Pipeline (https://www.ncbi.nlm.nih.gov). For the functional assignment of genes into COG categories, we utilized the eggNOG database (46). The replicon plot was generated using Proksee (https://proksee.ca/).

### Comparative genome analyses

The genomic sequence of 29 strains of *S. meliloti* with closed genomes (May 2023) was downloaded from the NCBI assembly database. The Get_homologues pipeline was used for comparative genomic analyses (47). The orthologous genes were defined using the default parameters. The core category included genes that were present in all genomes, the soft-core category included genes found in 95% of genomes, and the cloud category represented genes that were present in at least 2% of genomes. All genes not classified into the previous categories were part of the shell category. Figures showing shared and unique genes were created through the website: https://www.bioinformatics.com.cn/srplot. The prediction of phage-related elements such as prophages was performed using the PHASTER web server (48) and Phigaro (49). The phage sequences were reannotated using the PhageScope website (https://phagescope.deepomics.org/workspace). Insertion sequences were determined by ISEScan (version 1.7.2.2) (https://github.com/xiezhq/ISEScan). Clinker, a Python-based tool (https://github.com/gamcil/clinker), was used to visualize gene cluster comparisons.

### Phylogenetic analyses

For the construction of the VirB4/TrbE and TraA/MobZ phylogenetic trees, the proteins were aligned with the program of CLUSTAL Omega (50). Phylogenetic inference and model selection were performed simultaneously using IQ-TREE 1.6.12 (51) with support assessed via 1,000 ultrafast bootstrap replicates. The VT + F + G4 and LG + F + G4 amino acid substitution models were identified as the best-fitting models for TraA/MobZ and VirB4/TrbE data sets, respectively.

The accession numbers for the proteins selected for the TraA/MobZ and VirB4/TrbE phylogenies are listed in Tables S2 and S3, respectively, at https://doi.org/10.24215/10915datasetDBEERZ.

### Bacterial mating

Bacterial mating assays were performed as described by Simon et al. (52). Briefly, liquid cultures of donor and recipient cells were grown at 28°C to the early exponential phase and late exponential phase, respectively. Donor and recipient cells were mixed in a 1:1 volume ratio in a microcentrifuge tube and then concentrated by an 8-min centrifugation at 640 × *g*. The mating mixture was plated on TY plates and then incubated overnight at 28°C. Finally, the transconjugants and controls were removed and resuspended in 1 mL of TY and plated on selective TY medium supplemented with the appropriate antibiotics.

### DNA manipulation and genetic constructs

The total DNA and plasmid preparations, restriction-enzyme analysis, cloning procedures, and *E. coli* transformation were performed according to previously established techniques (53). PCR amplifications were carried out with recombinant Taq DNA polymerase or with PFU DNA polymerase as specified by the manufacturers. Amplicons were visualized in 1% agarose gels stained with ethidium bromide. A 100 bp ladder from PBL was used as a molecular weight marker. Primers used in this work are listed in Table S4 at https://doi.org/10.24215/10915datasetDBEERZ.

### Tagging of pSmeLPU88c

For construction of strain LPU88 (pSmeLPU88c-Nm), an intergenic region of 183 bp from pSmeLPU88c was amplified between the RP965_22845 and RP965_22850 using PFU DNA polymerase and the *F_pSymA88_ins/R_pSymA88*_*ins* primers. This fragment was cloned into the *SmaI* site of pK18mobsacB, yielding pK18mobsacB::ins. To introduce pK18mobsacB::ins into *S. meliloti* LPU88, the pK18mobsacB::ins was transformed into *E. coli* S17-1 and then conjugated with the LPU88 strain by biparental mating. The single recombinants were selected as Sm^R^ Nm^R^. To verify the insertion, PCR was performed with *F_cheq_pSymA88/m13fw-40* primers, and an expected 427 bp fragment was obtained.

### RNA extraction and RT-PCR

*S. meliloti* LPU88 and 1021 strains were grown in TY medium without antibiotics at 28°C and then scaled up to 25 mL of TY for growth to the mid-log phase (OD_600_ = 0.3–0.5). Cells were then divided into three equal portions and harvested at 4°C for 5 minutes. The resulting pellets were stored at –80°C. Total RNA was isolated according to the protocols provided by the manufacturer of TransZol (TransGen Biotech Co., Ltd.) and quantified using NanoDrop. Then, the total RNA was treated with DNase I at 37°C for 1 hour. Subsequently, cDNA was synthesized using the M-MLV reverse transcriptase protocol (PBL). The expression of the *rctA* gene was evaluated by PCR using gene-specific primers (*F_rctA/R_rctA*). Additionally, the expression of the Mpf/type IV secretion system (T4SS) machinery encoded on pSymA_1021_ and pSmeLPU88c and regulated by RctA (Mpf type II) was analyzed using specific primers (*F_trbE_1/R_trbE_1*). The size of the expected amplicon was 411 bp for both strains 1021 and LPU88. The expression of the second Mpf/T4SS system located on pSmeLPU88c (T4SSb) was also assessed using primers *F_trbE_2/R_trbE_2*. Finally, the expression levels of the Mpf/T4SS cluster identified on pSmeLPU88a (Mpf type IVb) were evaluated by PCR using primers *F_trbE_a/R_trbE_a*. The band sizes were 458 bp for *trbE_2* and 300 bp for *trbE* present in pSmeLPU88a.

### Curing of pSmeLPU88a

To eliminate pSmeLPU88a, the 1,370-bp fragment containing the incompatibility region *parS* of pSmeLPU88a was cloned into the replicative plasmid pBBRMCS-5, resulting in the construction of pBBRMCS-5::parS-pSmeLPU88a. *E. coli* S17-1 carrying this plasmid was then conjugated into *S. meliloti* LPU88, and the resulting transconjugants were analyzed using *in situ* lysis gel electrophoresis (54). Finally, *S. meliloti* LPU88 clones lacking plasmid pSmeLPU88a were tagged on pSmeLPU88c using the methodology described in “Tagging of pSmeLPU88c,” above.

### Construction of the *S. meliloti* LPU88 *rctA* mutant

A 97-bp internal fragment of the *rctA* gene was amplified by PCR with the primers *F_mut_rctA* and *R_mut_rctA*. The PCR fragment was then cloned into the pG18mob plasmid (Gm^R^) to generate pG18mob::rctA. The resulting plasmid was transferred by conjugation to strain LPU88ΔA to yield the pSmeLPU88c-rctA::pG18mob plasmid by site-specific insertional mutagenesis. The correct plasmid integration was confirmed by PCR using an external primer.

## RESULTS AND DISCUSSION

### Whole-genome sequencing and analysis

The genome sequence of *S. meliloti* LPU88 was obtained using short (Illumina) and long reads (ONT). A paired-end library was constructed and sequenced using the Illumina platform, which generated 3,387,162 reads. Similarly, a nanopore library was constructed and sequenced on the MinION platform to generate 147,731 reads with a total read length of 1,903,824,382. After Flye assembly and Pilon polishing, a complete circularized genome sequence of *S. meliloti* LPU88 was generated, with an estimated genome coverage of 270-fold. Data on the genome characteristics of strain LPU88 are presented in Table 1.

**TABLE 1:** *Sinorhizobium meliloti* LPU88 genome features

Feature	Chromosome	pSmeLPU88a	pSmeLPU88b	pSmeLPU88c (pSymA-like)	pSmeLPU88d (pSymB-like)
Size (bp)	3,827,946	182,781	35,933	1,361,493	1,600,864
GC content (%)	62.71	59.16	58.55	60.35	62.63
Total number of genes	3,724	174	32	1,329	1,470
rRNA operons	3	0	0	0	0
tRNA	52	0	0	1	1
Protein-coding genes	3,659	174	32	1,328	1,469
Genes with predicted function	3,170	134	28	1,134	1,335

**Fig 1:**
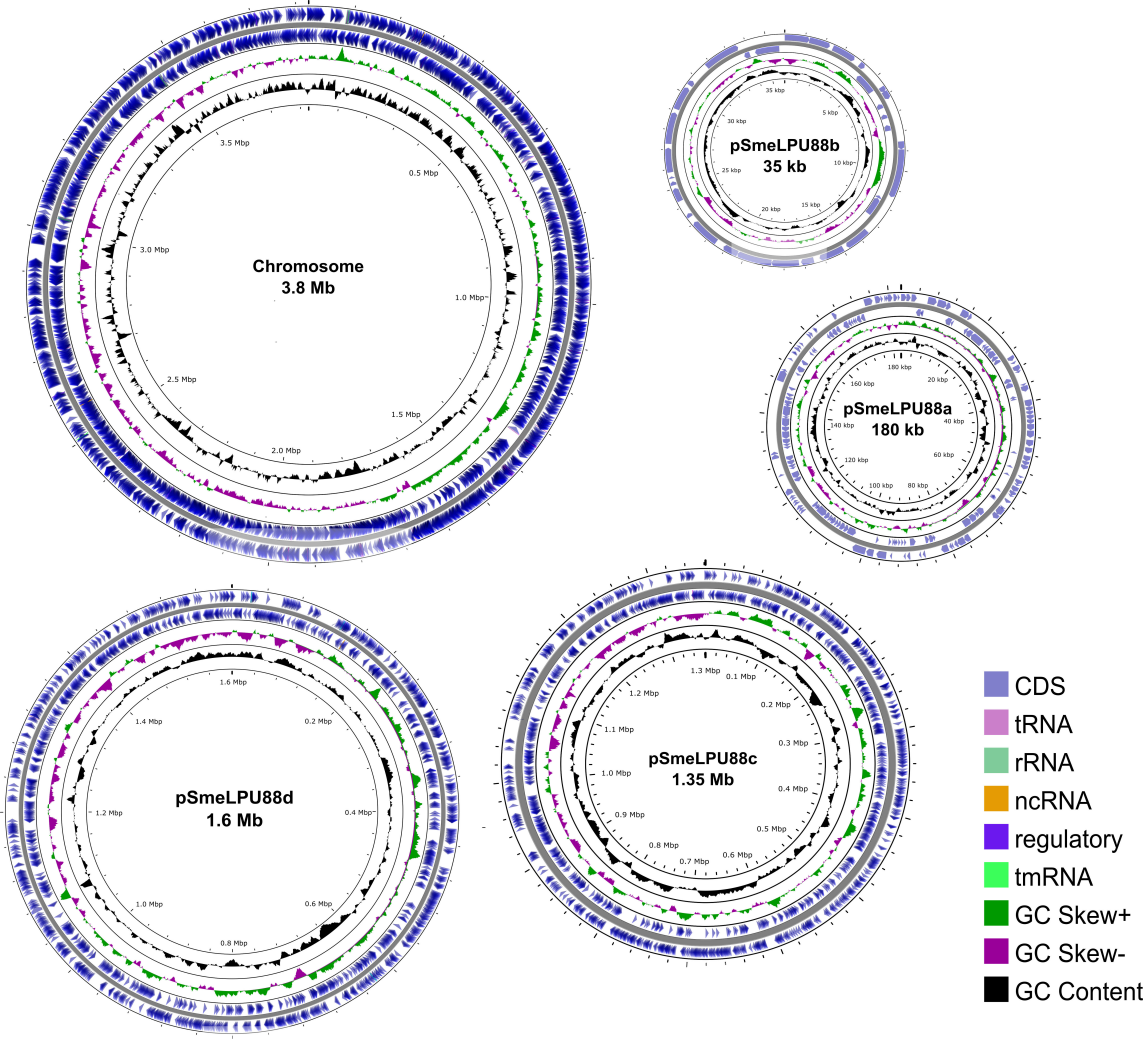
Genome map of *S. meliloti* LPU88. From the inner to the outer circle: GC content, GC skew (green and purple colors correspond to higher and lower than average GC skew, respectively), and predicted protein-coding sequences (CDSs). The plot was made using https://proksee.ca/.

The total genome size of LPU88 is 7,008,679 bp, with an average GC content of 62.1%. The genome size and GC content of LPU88 both fall within the expected ranges of 6.14–8.94 Mbp and 61.70%–62.40%, respectively, as observed in the 281 sequenced *S. meliloti* genomes deposited at the NCBI (https://www.ncbi.nlm.nih.gov/datasets/genome/?taxon=382). The genome consists of five circular replicons corresponding to a chromosome (3,827,946 bp); two megaplasmids, pSmeLPU88d (pSymB-like, 1,600,864 bp) and pSmeLPU88c (pSymA-like, 1,361,493 bp); and two accessory plasmids, pSmeLPU88a (182,781 bp) and pSmeLPU88b (35,933 bp). The number and size of replicons are consistent with the results obtained by an *in situ*-lysis gel electrophoresis analysis previously reported by Pistorio et al. (42). Moreover, the size of the conserved replicons of strain LPU88 was among the values reported in other rhizobial strains. The characteristics of the pSmeLPU88b plasmid were recently described by Luchetti et al. (41). A circular map of the complete replicons of LPU88 was generated through Proksee (55) (Fig. 1). With the genome sequence, the taxonomic classification of strain LPU88 was verified. We performed phylogenetic analyses using TYGS for whole genome sequences (56) and ANIm. The obtained values confirmed LPU88 as an *S. meliloti* strain (DDH > 85% and ANIm > 98%).

### Functional annotation and comparative genomics

**Fig 2:**
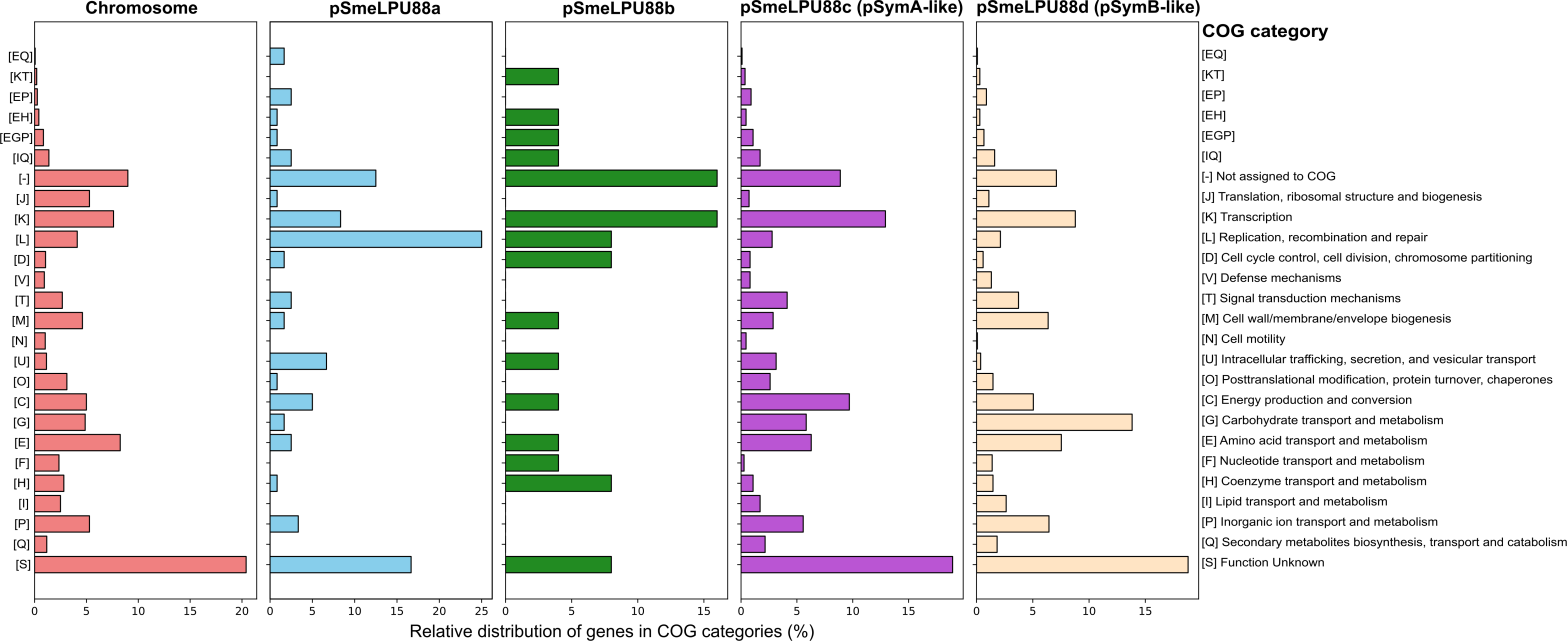
COG distribution of the protein-coding genes in the LPU88 genome. The panels represent the five replicons of LPU88, from left to right: chromosome, pSmeLPU88a, pSmeLPU88b, pSmeLPU88c, and pSmeLPU88d. Values are shown as the percentage of genes on each replicon belonging to each COG. Categories with two letters indicate that the gene product is associated with both functional categories.

After annotation, 3 rRNA operons, 54 tRNA genes, and 6,662 protein-coding sequences (CDSs) were predicted in the genome. A total of 5,801 CDSs were assigned to putative functions, while 861 were predicted to encode hypothetical proteins. The distribution of genes in COG categories for each replicon was evaluated using the eggNOG database (Fig. 2) (46). Similar to other sequenced *S. meliloti* strains, the COG categories S (function unknown), K (transcription), and genes not associated with any category were overrepresented. Additionally, the chromosome was enriched in the COG categories E (amino acid transport and metabolism), J (translation, ribosomal structure, and biogenesis), and P (inorganic ion transport and metabolism), as it occurs in other *S. meliloti* strains. The pSmeLPU88c was enriched in the functional categories C (energy production and conversion) and E (amino acid transport and metabolism). Essential genes for the establishment of a full symbiotic interaction (*nod*, *nif,* and *fix*) in the *S. meliloti* LPU88 genome were found in the symbiotic plasmid pSmeLPU88c (pSymA-like), as expected. The arrangement and genomic neighborhood of these genes are like those in *S. meliloti* strain 2011 (see Fig. S1 at https://doi.org/10.24215/10915datasetDBEERZ). Conversely, genes related to carbohydrate transport and metabolism (G), amino acid transport and metabolism (E), and cell wall/membrane envelope biogenesis (M) were overrepresented in the plasmid pSmeLPU88d (pSymB-like). This plasmid carries genes for surface polysaccharides that play an essential role during the infection stage (5).

Comparisons with the get_homologues pipeline of all 30 closed genomes of *S. meliloti* strains showed a core genome set of 2,363 orthologous genes: 1,391 in the chromosome, 664 in the pSymB-like plasmid, and 225 in the pSymA-like plasmid. The distribution of genes from the whole genome and each pSym into core, soft core, shell, and cloud categories (see “Comparative genome analyses,” above, for definitions) is shown in Fig. S2 to S4 at https://doi.org/10.24215/10915datasetDBEERZ. The percentage of core and soft core resulted in 87% for the chromosome, 83% for pSymB-like, and 45% for pSymA-like, corroborating the previous results, where pSymA-like plasmids showed a higher degree of variability (57). *S. meliloti* LPU88 contains at least 149 unique genes, distributed among the chromosome (52 genes), pSmeLPU88d (4 genes), pSmeLPU88c (46 genes), and the accessory plasmids. Of these, 105 genes were assigned to COG categories, whereas the remaining genes encoded hypothetical proteins. The presence of a substantial number of unique genes, particularly on plasmids, highlights the genetic plasticity of strain LPU88 and suggests ongoing acquisition of strain-specific functions. The distribution of the classified genes across COG categories is shown in Fig. S5 at https://doi.org/10.24215/10915datasetDBEERZ. Interestingly, on plasmid pSmeLPU88a, three clusters of 5, 6, and 15 genes were identified. However, no function could be associated with the clusters, since most of the genes were annotated as hypothetical proteins.

### Presence of mobile genetic elements

Like other *S. meliloti* strains, LPU88’s genome contains several genes encoding mobile genetic elements like phages, IS, and group II introns. To explore the rich diversity of genetic elements driven by horizontal gene transfer, the presence of phage sequences and insertion sequences was evaluated. PHASTER algorithm (48) and Phigaro (49) were used to detect the presence of phage sequences. A total of 14 regions were identified by PHASTER: 7 in the chromosome, 3 in each symbiotic plasmid, and 1 in pSmeLPU88a (see Table S5 at https://doi.org/10.24215/10915datasetDBEERZ). Five of the seven chromosomal regions found by PHASTER were also identified by Phigaro. These correspond to three regions that the software catalogs as “intact phages” (score assigned >90) and two assigned as questionable by the score obtained using PHASTER (score 70–90).

The unique phage sequence predicted on plasmid pSmeLPU88a was classified as “intact” by both PHASTER and Phigaro and assigned to the *Caudoviricetes* family. However, this predicted prophage shows inconsistencies between the two prediction tools, particularly in terms of size, and is considerably smaller than what would be expected for an intact member of the *Caudoviricetes* family. Indeed, the region lacks key structural and lysis-associated genes, further suggesting that it does not represent a complete functional phage.

In contrast, the phage sequences detected in pSmeLPU88c and pSmeLPU88d were only identified by PHASTER and classified as incomplete. Additionally, “intact” phage sequences detected with PHASTER were re-annotated using PhageScope, with the three “intact” phage sequences on the chromosome showing the highest annotation quality. In all cases, genes encoding for typical phage proteins, such as integrases and genes associated with capsid packaging and structural proteins, were detected (see Fig. S6 at https://doi.org/10.24215/10915datasetDBEERZ). To assess whether the number of “intact” phages observed is common among other *S. meliloti* strains, we extended the PHASTER analysis to the 29 closest genomes. A total of 58 “intact” phage sequences were identified across 21 genomes (see Table S6 at https://doi.org/10.24215/10915datasetDBEERZ). Among these, 42 were located on the chromosome, 9 on pSymA, 6 on accessory plasmids, and 1 on pSymB. However, six of these regions were considerably smaller than expected for complete phages, lacked key structural genes, and are therefore likely artifacts of the prediction algorithms rather than functional prophages. The genome of strain USDA1021 exhibited the highest number of phages in its genome (seven). Interestingly, several strains—including AK21, AK83, M270, RCAM1115, RU11001, and USDA1021—harbor at least three “intact” phage regions on the chromosome, similar to strain LPU88. We found that most of the detected phages were related to those associated with the genera *Rhizobium*, *Mesorhizobium*, and *Sinorhizobium*, with PHAGE_Sinorh_phiLM21_NC_029046 being the most common phage (21 matches).

The identification of IS elements present in the different replicons of strain LPU88 was made with ISEScan (58), and the data are presented in Table S7 at https://doi.org/10.24215/10915datasetDBEERZ. In total, 105 IS elements belonging to 13 families were identified, distributed across the five replicons: 33 in the chromosome, 14 in pSmeLPU88d, 35 in pSmeLPU88c, 20 in pSmeLPU88a, and 3 in pSmeLPU88b. The most abundant families of IS elements were IS110 (21), IS256 (17), ISNCY (15), and IS66 (14) (see Fig. S7 at https://doi.org/10.24215/10915datasetDBEERZ). While the ISNCY family was found in all five replicons, IS110 was found in all but pSmeLPU88a, and ISL3, IS6, and IS5 were only found in pSmeLPU88a. Extending the analysis of ISs to the remaining genomes examined revealed that members of the IS110, IS256, IS3, IS30, IS4, IS481, IS630, IS66, and ISNCY families are present in all genomes (see Table S8 at https://doi.org/10.24215/10915datasetDBEERZ). Analysis of their distribution across replicons showed that IS4 and IS481 are overrepresented on chromosomes, whereas IS3, IS30, IS66, and ISNCY are overrepresented on pSymA-like plasmids (see Fig. S8 at https://doi.org/10.24215/10915datasetDBEERZ).

Additionally, group II introns have been found in the *S. meliloti* genome. Within these, RmInt1 is one of the most studied introns and is widely distributed in strains of both *S. meliloti* and *Sinorhizobium medicae* (59). RmInt1 is a group II intron that is present in a wide variety of *S. meliloti* strains. These sequences are found distributed across various replicons such as the chromosome, pSymA, pSymB, and in accessory plasmids (60). In strain LPU88, multiple sequences related to RmInt1 were identified. In strain LPU88, four sequences related to RmInt1 were identified. Three copies showed 99% identity to RmInt1 and were located on pSmeLPU88d, pSmeLPU88c, and the accessory plasmid pSmeLPU88a. The remaining sequence, located on pSmeLPU88c, displayed 89% identity and was similar to the RmInt1 intron sequence previously described on plasmid pRmeGR4b of strain GR4 (59).

Overall, the significant number and variety of mobile genetic elements found in the LPU88 strain underscore the dynamic nature of the LPU88 genome.

### Replication characteristics of LPU88 plasmids

**Fig 3:**
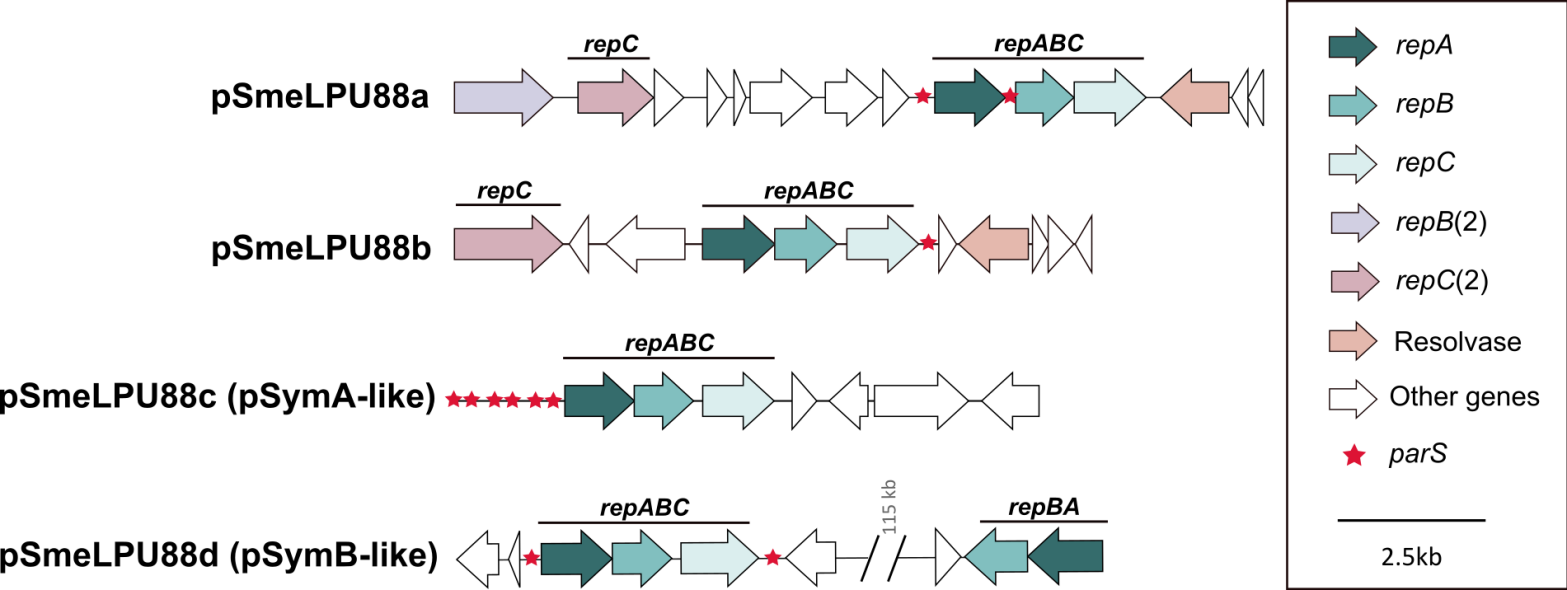
Distribution of replication modules in pSmeLPU88a, pSmeLPU88b, pSmeLPU88c (pSymA-like), and pSmeLPU88d (pSymB-like) from *S. meliloti* LPU88. The colored arrows represent the genes involved in replication, and the red stars indicate the *parS* sites.

The presence of large amounts of plasmidic replicons is a common feature of the *Sinorhizobium* genus. The *repABC*-type replicons, which predominate in rhizobial plasmids, consist of four components: a three-gene operon (*repA, repB,* and *repC*) and a small regulatory antisense RNA involved in incompatibility (23, 24). RepC is the initiation protein (61), and the interaction of RepA, RepB, and *parS* (centromere-like sequence) conforms to the plasmid partitioning machinery (23, 62, 63). Other rhizobial origins of replication belong to the *repC* family, which lacks the *repA* and *repB* genes but is evolutionarily related to the *repABC* family (25, 64). In the *S. meliloti* LPU88 genome, six *repC* genes were identified: one in each of the pSyms and two in each of the accessory plasmids. Four of these genes belong to the *repABC* family, while the other two belong to the *repC* family (Fig. 3).

We have previously identified and reported both types of replicases in pSmeLPU88b (41). Similarly, the pSmeLPU88a plasmid contains two replication modules: one belonging to the *repABC* family and the other to the *repC* family (Fig. 3). There is an additional *repB* gene (*repB_2_*) in pSmeLPU88a, which is longer (1.9 kb) compared to those found in the *repABC* operons (1 kb). This *repB_2_* gene presented different conserved domains (PRK13832, TIGR00180, cd_16406) than those of the *repB* in the *repABC* operon (PRK13866, TIGR03454, cd_16405). Additionally, the fact that the intergenic region between *repB_2_* and *repC_2_* is longer (573 bp compared to approximately 160 bp) suggests that they may not be forming an operon. Similar genomic structures were observed in the paccessoryA, a plasmid of strains HM006 and T073 of *S. meliloti,* and in pSF45436e from *Sinorhizobium fredii* CCBAU45436 (41).

MacLellan et al. (65, 66) characterized the *repABC* features of pSymA of strain *S. meliloti* 2011, finding six *parS* sites upstream of the *repA* gene (see Fig. S9 at https://doi.org/10.24215/10915datasetDBEERZ). We compared the sequence of the pSmeLPU88c *repABC* operon to that of the *repABC* of the closed pSymA-like plasmids and found that the three coding genes, as well as the antisense RNA, presented a high degree of sequence identity. The exceptions were the *repABC* present in pSymA_AK83_ and pSymA_L6-AK89_, which showed a divergent ctRNA and less conserved sequence for *repABC* genes (see Fig. S9 at https://doi.org/10.24215/10915datasetDBEERZ). The *parS* sequence analysis revealed variations in some of the pSymA-like plasmids compared to those described in pSymA_2011_ (see Fig. S9C at https://doi.org/10.24215/10915datasetDBEERZ). However, palindrome 1, responsible for the incompatibility effects (66), was conserved in almost all plasmids except for pSymA_AK83_. The pSmeLPU88d plasmid exhibited a *repABC* family origin of replication, which was conserved among other pSymB-like plasmids.

### Characteristic of conjugation modules identified in LPU88 plasmids

**Fig 4:**
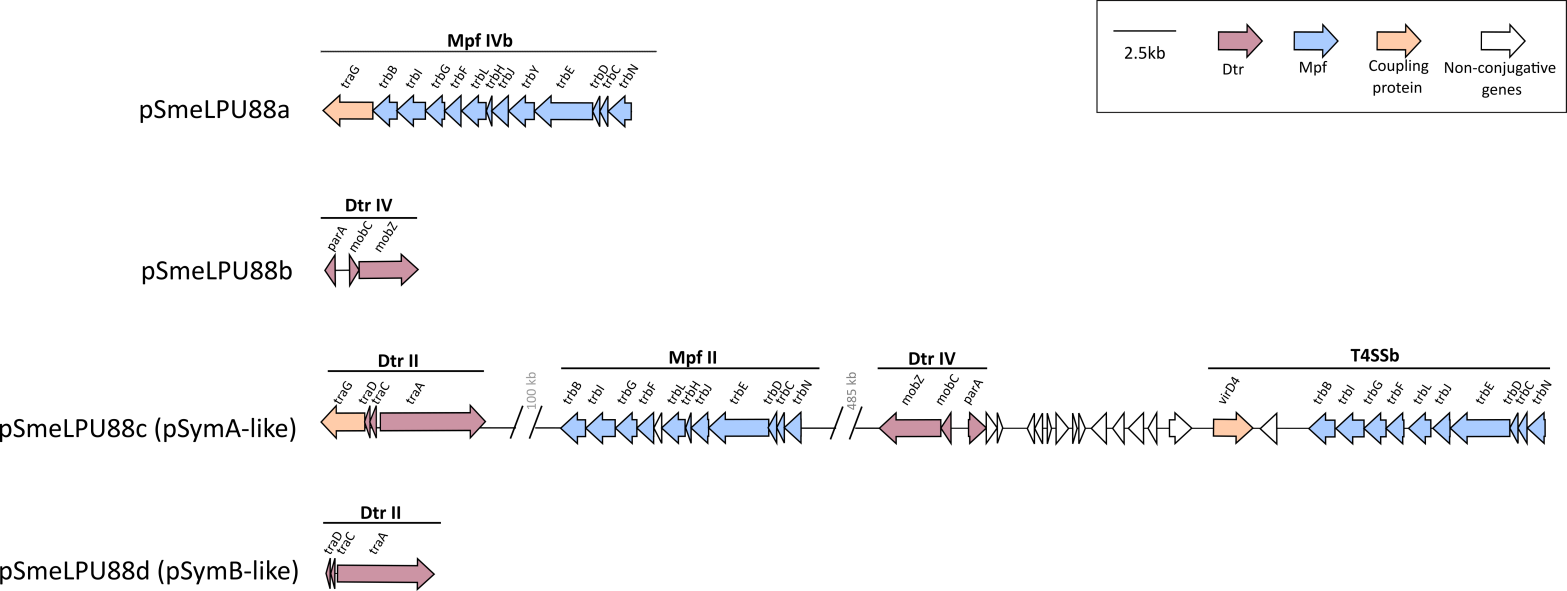
Conjugation systems present in the LPU88 genome, with classification obtained from the phylogenetic trees of TraA/MobZ and TrbE/VirB4. Genes associated with Dtr, Mpf, and the coupling protein are shown with different colors.

**Fig 5:**
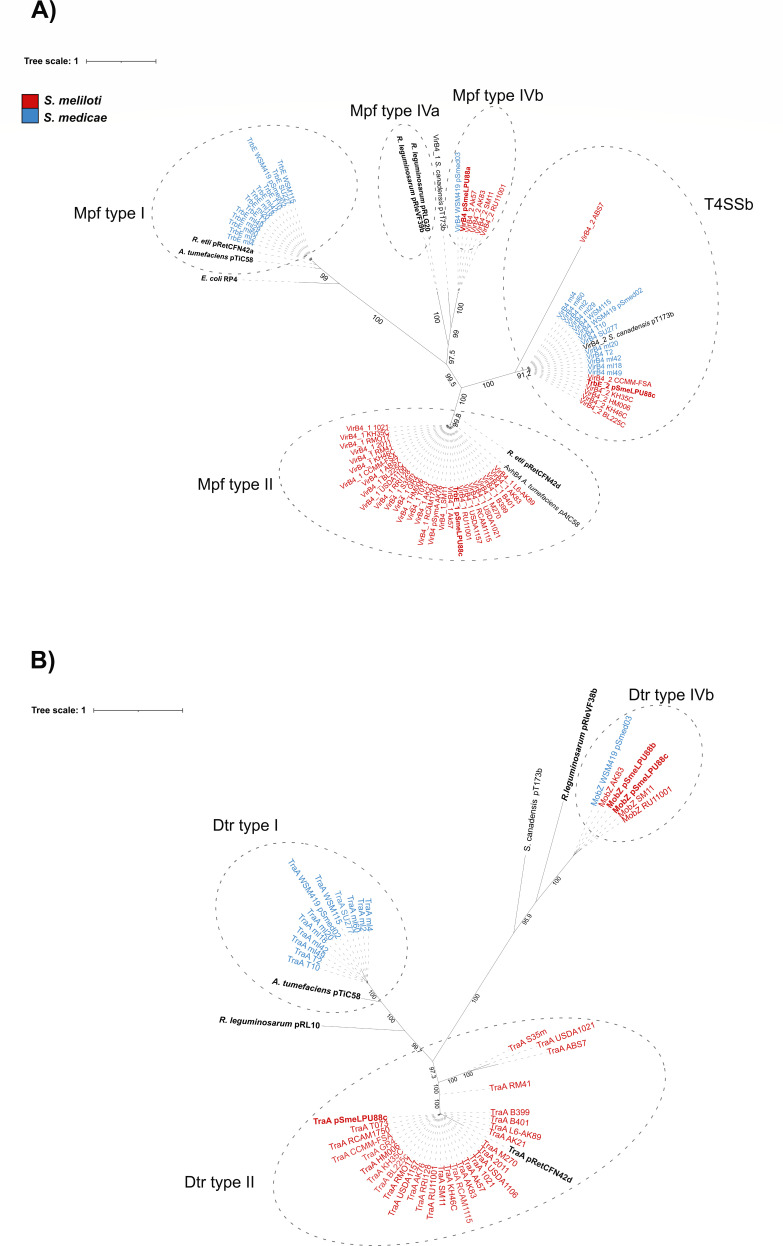
Phylogenetic analysis of TrbE/VirB4 proteins (**A**) and TraA/MobZ (**B**) relaxases in pSymA-like plasmids of *S. meliloti* and *S. medicae* strains. The conjugation systems present in the accessory plasmids of LPU88 were included. Trees were constructed based on protein sequence alignments created with ClustalX, employing the ML method with 1,000 bootstrap replicates. Red color indicates the sequences from *S. meliloti,* and blue color indicates the sequences from *S. medicae*.

In many rhizobial strains, plasmids are transferred through conjugation (67–71). For such transfer, the Mpf genes (designated *trb* or *vir*) encode a T4SS, which establishes contact among the cells, while the Dtr genes (*tra* or *mob*) are involved in the processing and replication of the DNA. In addition, since transfer requires coupling of Dtr and Mpf systems, a third component needed is the coupling protein. The genomic location of the coupling protein could be associated with either Mpf or Dtr. In previous studies, the different conjugation systems present in rhizobia were classified into four groups based on the relaxase phylogenetic tree and the syntenic organization of Dtr and Mpf (27–29). To investigate the number and characteristics of the Mpf/Dtr present in the plasmids of *S. meliloti* strain LPU88, we made a blastp search using TrbE/VirB4 to identify Mpf/T4SS and TraA/MobZ for Dtr using the sequence of *S. meliloti* 2011 strain as reference. We identified three Mpf/T4SS and four Dtr systems (pSmeLPU88a, one Mpf; pSmeLPU88b, one Dtr; pSmeLPU88c, two Mpf, two Dtr; and pSmeLPU88d, one Dtr; Fig. 4). The identified systems were classified by the construction of phylogenetic trees (Fig. 5). The Mpf/T4SS system found in pSmeLPU88a belongs to the IVb group, similarly to those identified in the pSymA-like plasmids of *S. meliloti* strains SM11, RU11001, Ak57, and AK83. This Mpf found on pSmeLPU88a is conjugatively functional, since it was previously described as a helper for mobilizing pSmeLPU88b (42). Conversely, the other accessory plasmid pSmeLPU88b presents only a Dtr system (no Mpf mapped), which was previously characterized by Giusti et al. (29).

Results also showed a Dtr system on pSmeLPU88d, with high DNA sequence identity with other Dtr systems present in pSymB-like replicons (72). The pSymB_1021_ could be mobilized by the type IV secretion system encoded by pSymA (72) and uses its own relaxases to achieve the highest transfer efficiency (72).

The remaining Mpf/T4SS and Dtr systems were mapped on pSmeLPU88c (Fig. 4). One pair of these, Mpf/T4SS and Dtr, belongs to the type II group according to Ding and Hynes classification (27, 28) and presented synteny with the ones on pSymA_1021_. Pérez-Mendoza et al. (31) described that the conjugation of pSymA_1021_ was regulated by the repression mediated by RctA and activated by RctB. The expression of *rctB* requires the inhibition of the transcriptional repressor RctR, which does not directly repress *rctB* transcription (34). RctR represses the transcription of an adjacent operon that encodes several enzymes, while the *rctC*-coded protein activates the transcription of the antirepressor *rctB* (34). The search for this regulatory circuit in pSmeLPU88c and the comparison to closed pSymA-like plasmids showed interesting results. All plasmids have the repressor *rctA*; however, only 13 out of 30 pSymA-like plasmids possess the activation circuit (see Fig. S10 at https://doi.org/10.24215/10915datasetDBEERZ). These findings could have important consequences on the conjugative behavior of pSymA-like plasmids since they may behave as non-transferable unless another uncharacterized activation mechanism is present. However, the phylogenetic analysis revealed that the Mpf/T4SS type II group of *S. meliloti* is related to the Mpf/T4SS present in pAtC58 plasmid from *Agrobacterium tumefaciens* C58 (Fig. 5A), which is implicated in the conjugative transfer of the cryptic plasmid (73) and does not present the regulatory circuit.

The other Dtr in pSmeLPU88c belongs to the Type IVb group (Fig. 5B). The *parA–mobZ* arrangement in pSmeLPU88c was syntenic with the Dtr found on pSmeLPU88b plasmid (Fig. 4), with the *parA*-like gene longer than pSmeLPU88c’s, and the MobZ_pSmeLPU88c_, with 90.29% identity with the MobZ_pSmeLPU88b_. The pSymA-like plasmids of strains AK83, SM11, and RU11001 also contain an additional Dtr system similar to that observed in pSmeLPU88c, sharing 82.77% and 90.91% sequence identity with *mobZ* and *mobC* of pSmeLPU88b, respectively.

Finally, the second Mpf/T4SS found on pSmeLPU88c, along with five other pSymA-like plasmids (KH46C, BL225C, HM006, CCMM-FSA, and KH35c), was not included in any of the classification groups previously described by Ding and Hynes (27).

However, according to Sugawara et al. (74), it belongs to the T4SSb group. Nelson et al. (75) determined that the T4SSb system present in strain KH46C is involved in symbiosis with *Medicago* species through its association with effector proteins. The authors have not identified the replicon that bears the T4SS, although we were able to map such a system in the pSymA-like plasmid of strain KH46C. Overall, these results suggest that the system has a potential effect on the symbiotic process rather than in conjugation.

### Analysis of Mpf/T4SS and Dtr systems from pSymA-like plasmids in *Sinorhizobium*

In *Sinorhizobium,* recombination significantly influences genetic diversity and adaptation (76). It appears to occur preferentially within and among the megaplasmids of *Sinorhizobium* species (76). To better understand the pSymA-like conjugative behavior within the *Sinorhizobium* species, we compared the T4SS and the Dtr of the pSymA plasmids of *S. meliloti*, *S. medicae,* and other *Sinorhizobium* species. The results showed a similar topology between the phylogenetic trees corresponding to the TrbE/VirB4 homologs (Fig. 5A) and TraA/MobZ (Fig. 5B). In both trees, it was possible to observe well-defined groups of proteins. Two groups in each tree correspond to plasmids either of *S. medicae* (Type I) or *S. meliloti* (Type II). In the tree of TrbE/VirB4, one group corresponds to proteins that do not belong to any of the conjugal systems but were associated with the translocation of effector proteins (T4SSb) (74, 75). This group comprises proteins from both *S. meliloti* and *S. medicae* yet exhibits species-specific subclustering (Fig. 5A). Another group in both trees included *Sinorhizobium* proteins belonging to group IVb, either TrbE/VirB4 or TraA/MobZ. Interestingly, in the newly identified *Sinorhizobium canadensis* strain (77), the TrbE and TraA proteins encoded by the pT137b plasmid do not cluster within either of the two previously defined type IV subgroups (Fig. 5).

### Functional characterization of the conjugation properties of pSmeLPU88c

Although the additional Mpf/T4SS of pSmeLPU88c was related to the one found on *S. meliloti* KH46C (T4SSb), which is involved in effector protein transport, a Dtr system was identified near this secretion system (Fig. 4). Thus, the finding of an additional Mpf/T4SS and Dtr on plasmid pSmeLPU88c prompted us to investigate whether these regions could produce the conjugative transfer of the symbiotic plasmid pSmeLPU88c. To this end, we constructed a single integration vector for tagging pSmeLPU88c, designated pK18mobsacB::ins. The plasmid was transferred to the strain LPU88, yielding LPU88 (pSmeLPU88c-Nm). The correct integration of the plasmid was examined by PCR using an external primer. Conjugation assays were performed using *A. tumefaciens* UBAPF2C (Cm^R^) as the recipient strain. Several Nm-resistant *A. tumefaciens* UBAPF2C transconjugants were obtained at a frequency of 8 × 10^−7^ transconjugants per recipient cell. Given that the transfer frequency is comparable to that of pSymA_1021_ under active conditions (34), we cannot exclude the possibility that pSmeLPU88c conjugation occurred via the type II conjugative system. To answer which system is responsible for the pSmeLPU88c transfer, we first characterized *in silico* if the type II system was identical to the one present in pSymA_1021_. The alignment of the *rctA* gene of strains LPU88 and 1021 revealed a 100% identity at the nucleotide level. In addition, the intergenic region of *rctA* and the *trb* operon showed 99% sequence identity, with the proposed RctA-binding site being totally conserved. Then, to determine whether the *rctA* gene is transcribed in the LPU88 strain background, a PCR was performed using cDNA as a template, with cDNA from the 1021 strain included as a control. The analysis confirmed that *rctA* is transcribed in LPU88 (see Fig. S10A at https://doi.org/10.24215/10915datasetDBEERZ). To further investigate which of the Mpf/T4SS systems is responsible for the conjugal transfer of pSmeLPU88c, we analyzed the expression of the three transfer systems present in LPU88 using the same cDNA. The result showed that the Mpf/T4SS on the pSmeLPU88a accessory plasmid was expressed, the T4SSb on pSmeLPU88c produced no signal, and RctA/RctB-regulated Mpf/T4SS presented a faint band (see Fig. S11B at https://doi.org/10.24215/10915datasetDBEERZ). Finally, to determine if the Mpf/T4SS of the pSmeLPU88a plasmid was responsible for the transfer of pSmeLPU88c, we used an LPU88 derivative lacking the accessory plasmid (LPU88ΔA), and no transconjugants were obtained as a result of the experiment (frequency < 10^−9^ transconjugants per recipient). To evaluate the functionality of the RctA/RctB system in the LPU88ΔA strain, we generated an insertional mutant of the *rctA* repressor and tested the conjugative behavior. As a result of the experiment, no transconjugants were observed. This result reinforces the idea that the Mpf from pSmeLPU88a is necessary for pSmeLPU88c mobilization.

Additionally, we evaluated whether the transfer of pSmeLPU88a to a strain without accessory plasmids (like 2011) could mediate the mobilization of a marked version of pSymA_2011_, which is not transferable under laboratory conditions on its own. However, under these conditions, no transconjugants of pSymA_2011_ were obtained (frequency < 10⁻⁹ transconjugants per recipient).

All these results allow us to conclude that most likely, the plasmid pSmeLPU88c was mobilized by the Mpf/T4SS of the pSmeLPU88a accessory plasmid, a behavior not previously described in pSymA-like plasmids of *S. meliloti*.

### Concluding remarks

In this work, we performed a comprehensive analysis of the *S. meliloti* LPU88 genome, emphasizing the structure and function of its plasmids. The complete genome sequence of LPU88 reveals a genomic architecture consistent with that of other *S. meliloti* strains, including GC content, genome size, and replicon organization.

Our comparative genomics study further elucidated the functional landscape of the LPU88 genome, showing overrepresented COG categories consistent with other sequenced *S. meliloti* strains (8). The analysis of the shared genes in each conserved replicon highlighted the role of the pSymA-like plasmid, not only in the symbiotic process but also in the saprophytic life, given its more diverse genetic contents. This supports the idea that pSymA-like plasmids contribute to the emergence of new functions (8) and are consistent with the high genetic polymorphism observed in pSymA (78).

Additionally, the identification of distinct Dtr and Mpf systems, especially in pSmeLPU88a and the pSymA-like plasmid pSmeLPU88c, underscores the evolutionary adaptations that promote plasmid transmission, stability, and potential roles in the spread of symbiosis. In addition, the analysis of conjugative elements in the pSymA plasmids of *S. meliloti* and *S. medicae* highlights the intricate evolution and variability of conjugation systems across *Sinorhizobium* species. In addition, the comparison of regulatory circuits across pSymA-like plasmids from *S. meliloti* reveals a heterogeneous distribution of activation circuits, suggesting differential evolutionary pressures among these plasmids.

Moreover, the presence of T4SSb on pSymA-like plasmids lacking Dtr systems suggests potential alternative functions for these systems, possibly related to the transport of effector proteins, as described by Chen et al. (73). In addition, we identified the Mpf/T4SS system responsible for the mobilization of pSmeLPU88c, which is located in the accessory plasmid pSmeLPU88a. This finding highlights the modular and dynamic nature of plasmid-mediated horizontal gene transfer and represents the cornerstone for further studies of the plasmid transfer in *Sinorhizobium*.

## Data Availability

Genome sequences are available in the NCBI database under the accession numbers CP135239, CP135240, CP135241, CP135242, and CP135243 for the LPU88 chromosome, pSmeLPU88a, pSmeLPU88b, pSmeLPU88c, and pSmeLPU88d, respectively.
